# Synaptically-Competent Neurons Derived from Canine Embryonic Stem Cells by Lineage Selection with EGF and Noggin

**DOI:** 10.1371/journal.pone.0019768

**Published:** 2011-05-17

**Authors:** Jared T. Wilcox, Jonathan K. Y. Lai, Esther Semple, Brigitte A. Brisson, Cathy Gartley, John N. Armstrong, Dean H. Betts

**Affiliations:** 1 Department of Biomedical Sciences, Ontario Veterinary College, University of Guelph, Guelph, Ontario, Canada; 2 Institute of Medical Science, University of Toronto, Toronto, Ontario, Canada; 3 Department of Clinical Studies, Ontario Veterinary College, Guelph, Ontario, Canada; 4 Department of Population Medicine, Ontario Veterinary College, Guelph, Ontario, Canada; University of Colorado, Boulder, United States of America

## Abstract

Pluripotent stem cell lines have been generated in several domestic animal species; however, these lines traditionally show poor self-renewal and differentiation. Using canine embryonic stem cell (cESC) lines previously shown to have sufficient self-renewal capacity and potency, we generated and compared canine neural stem cell (cNSC) lines derived by lineage selection with epidermal growth factor (EGF) or Noggin along the neural default differentiation pathway, or by directed differentiation with retinoic acid (RA)-induced floating sphere assay. Lineage selection produced large populations of SOX2+ neural stem/progenitor cell populations and neuronal derivatives while directed differentiation produced few and improper neuronal derivatives. Primary canine neural lines were generated from fetal tissue and used as a positive control for differentiation and electrophysiology. Differentiation of EGF- and Noggin-directed cNSC lines in N2B27 with low-dose growth factors (BDNF/NT-3 or PDGFαα) produced phenotypes equivalent to primary canine neural cells including 3CB2+ radial progenitors, MOSP+ glia restricted precursors, VIM+/GFAP+ astrocytes, and TUBB3+/MAP2+/NFH+/SYN+ neurons. Conversely, induction with RA and neuronal differentiation produced inadequate putative neurons for further study, even though appropriate neuronal gene expression profiles were observed by RT-PCR (including Nestin, TUBB3, PSD95, STX1A, SYNPR, MAP2). Co-culture of cESC-derived neurons with primary canine fetal cells on canine astrocytes was used to test functional maturity of putative neurons. Canine ESC-derived neurons received functional GABA_A_- and AMPA-receptor mediated synaptic input, but only when co-cultured with primary neurons. This study presents established neural stem/progenitor cell populations and functional neural derivatives in the dog, providing the proof-of-concept required to translate stem cell transplantation strategies into a clinically relevant animal model.

## Introduction

Cell-based therapeutics has received incredible interest for treating central nervous system diseases, and have been applied in many models of spinal cord injury (SCI) [1]. Cell transplantation strategies must be validated as safe and efficacious in preclinical models before their translation to humans. Unfortunately, several human clinical trials for CNS disorders have failed to show functional benefits, even after the intervention has shown significant, meaningful recovery in rodent models. This gap in translation has been seen with putative treatments for stroke, Alzheimer's disease and SCI [2], [3], [4], [5], [6], [7]. In light of this, many researchers have called for the development of large animal models to test preclinical trials for CNS disease, including SCI [1], [8], [9], [10]. The domestic dog has the greatest parity to humans in genetic diseases [11] with over 220 common genetic diseases (the Rhesus monkey boasts 5). Additionally, expert veterinary care offers pre- and post-operative assessment, real-time referral centre setting, treatment of spontaneous injury, and accurate extrapolation to human treatment trajectories. These factors have motivated scientists to call for canine cell transplantation models of SCI [12], [13].

Stem cell grafts have been generated for canine transplantation strategies with some myelinating cells types more thoroughly studied than in mice or humans [14], [15]. To date, strategies have been developed for canine SCI using mesenchymal stromal cells [16], [17]; autologous Schwann cells and olfactory ensheathing cells, including a phase I trial [18], [19]; and, human immortalized neural stem cells (NSCs) [20], [21]. While transplanting human-derived cells in canine models is valuable, it does not allow for testing and developing autologous or induced pluripotent stem cell (iPSC) transplantation in large animal models. Clinical relevance of the canine platform is further amplified given that dogs and humans share a common environment, leading to analogous SCI from motor vehicle collisions and disc degeneration [22], [23]. This allows for synergy between laboratory concept and clinical application with real-time realization.

Evaluating stem cell-derived transplantation in pre-human trials requires comparable protocols of isolating, manipulating and introducing graft tissue generated from pluripotent cell lines. To date, there have been very few self-renewing ESC lines generated from non-primate, non-rodent species [9], none of which have been shown capable of producing functional derivatives. Canine ESC lines have been established [24], [25], [26], however, proper manipulation of these cells to produce operative cell types has not been reported. Repair of the CNS will require neurons or oligodendroglial cells produced, preferably, from autologous sources. Reynolds and Weiss [27] were the first to isolate neural stem cells (NSCs) from fetal mice, using basic fibroblast growth factor (bFGF) and epidermal growth factor (EGF) to culture NSCs in a neurosphere (NS) assay. Definitive proof of neurogenesis was shown when electrophysiology was used to prove NSC-neurons from rodents and humans integrated into host tissue, and were synaptically competent [28], [29].

Neural stem cells can also be grown efficiently in monolayers, without cell-cell niche interactions in spheres, using the same EGF signaling to specify the neural lineage [30]. Human ESCs and iPSCs can also be restricted to the neural lineage by the administration of Noggin to repress BMP signaling [31], [32]. Furthermore, it has been shown that human ESCs maintained in the absence of mitogens and exogenous signals select the neural lineage fate, causing neural selection to be referred to as the default pathway [33]. This discovery has led to efficient and scalable production of purified NSC populations through lineage selection, avoiding previous methods that employed high-dose growth factors and mitogens such as retinoic acid (RA) [34]. Although multi-stage RA-based protocols are still used by several labs, there is controversy as to whether these protocols should be used to differentiate pluripotent and mesenchymal cells to neural fates [35], [36], [37], [38]. Nonetheless, transplantation of NSCs and glia-restricted precursors has been shown to greatly improve motor and sensory function in rats following SCI, and should be investigated further [39], [40], [41].

Here, we present NSC and primary fetal cerebral lines established in the dog. Canine (c)ESC-derived NSCs and primary lines were used to generate populations of neural progenitors and functional neurons. We used two types of cESC lines, derived from blastocyst explants that exhibit a neural default (OVC.EX), or derived from immunodissection of the ICM that do not exhibit a neural default (OVC.ID) [24]. We show that Noggin and EGF lineage selection of OVC.EX lines produces robust neural progenitors as well as large populations of mature neurons that exhibit proper immunophenotypes and spontaneous synaptic activity. We then tested lineage selection against directed neural differentiation using the non-neural OVC.ID lines and a standard RA-based floating sphere assay. The RA-based differentiation produced appropriate expression profiles; however, it did not produce neuronal morphology or sufficient number of putative neurons for analysis. To our knowledge, this is the first report of NSCs, primary fetal lines, and functional neurons derived in a domestic species. The domestic dog is an optimal model for the next phase of pre-human trials in SCI due to the availability of expert veterinary facilities, parity with human genetics and disease, and trauma occurring in shared environments [13]. By developing protocols to generate and manipulate potent cNSC lines, we hope to move cell transplantation to the veterinary centre and create clinical pre-human models of CNS damage and traumatic spinal cord injury.

## Results

### Canine explant (OVC.EX) ESC lines exhibit a neural default

**Figure 1:**
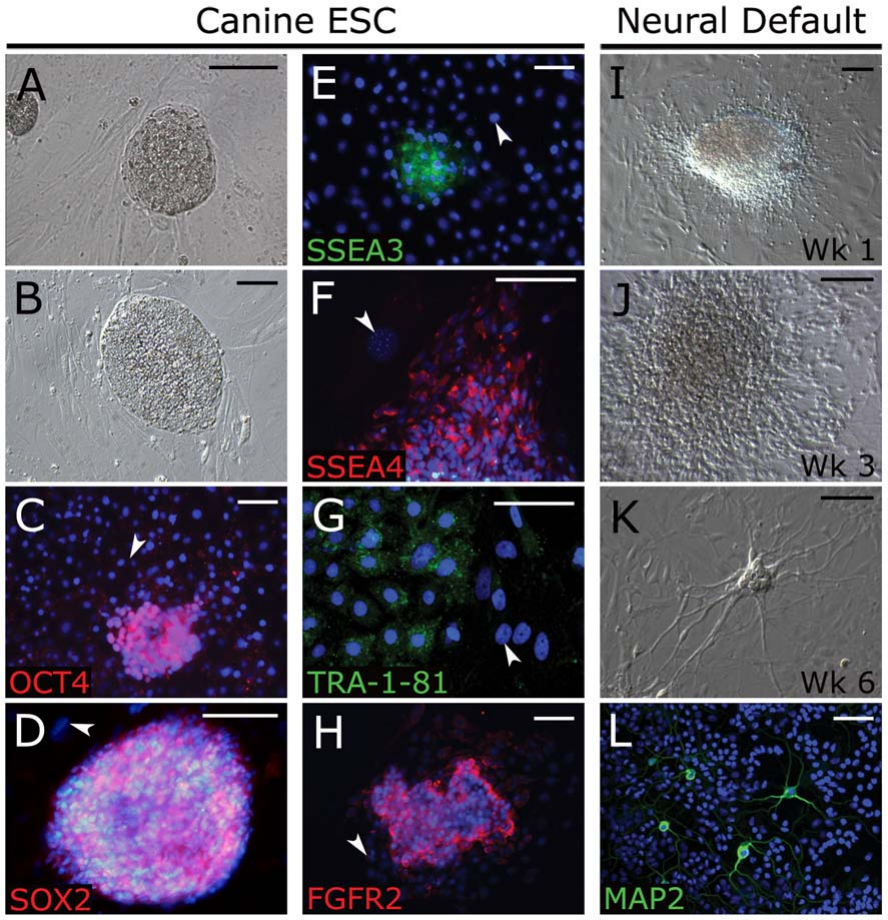
Canine ESCs express appropriate markers and neural lineage default differentiation. Canine ESCs derived from whole embryo explants cultured on MEFs in the presence of LIF and bFGF exhibit tightly packed, flat human ESC-like colonies (A, B), and express OCT4 (C), SOX2 (D), ESC surface markers SSEA 3 (E), SSEA4 (F), Tra-1-81 (G) and FGFR2 (H). Expression of ESC makers was not seen in MEF feeder cells (arrowhead, C–H). Upon removal of LIF/bFGF and addition of 10% knock-out serum, cESC colonies rapidly differentiate (I) with outgrowths of radial cells at weeks 2–3 (J), which produce neural morphologies at 4 weeks (K) that express neural-specific protein MAP2 (L). DAPI was used as nuclear counterstain (blue). Scale bar  =  100 µm. Abbreviations: cESC, canine embryonic stem cell; MEF, mouse embryonic fibroblast; Wk, week.

To determine the neural capacity of canine ESC lines, we used cell lines from explant (OVC.EX.4, 5, 7) and immunodissected (OVC.ID.20, 21, 23) embryos that we previously showed to be undifferentiated, self-renewing and pluripotent [24]. When cultured in LIF and bFGF, these cell lines produce tightly packed, flat colonies (Fig. 1A,B) that express ESC transcription factors OCT4 and SOX2 (Fig. 1C,D), surface glycoproteins SSEA3, SSEA4 and TRA-1-81 (Fig. 1E–G) and ESC niche-related receptor FGFR2 [42] (Fig. 1H). Canine ESC colonies from explant lines rapidly differentiate and exhibit outgrowths of bipolar cells when grown in serum but without LIF, FGF2 or MEF feeders for 1 week (Fig. 1I). These colonies produce diffuse monolayers of migrating cells by 3 weeks (Fig. 1J), and derive cells with multiple MAP2+ processes by 4 weeks (Fig. 1K, L).

### RA-induced differentiation generates incomplete neural determination from immunodissected (OVC.ID; non-neural default) cESC lines

**Figure 2:**
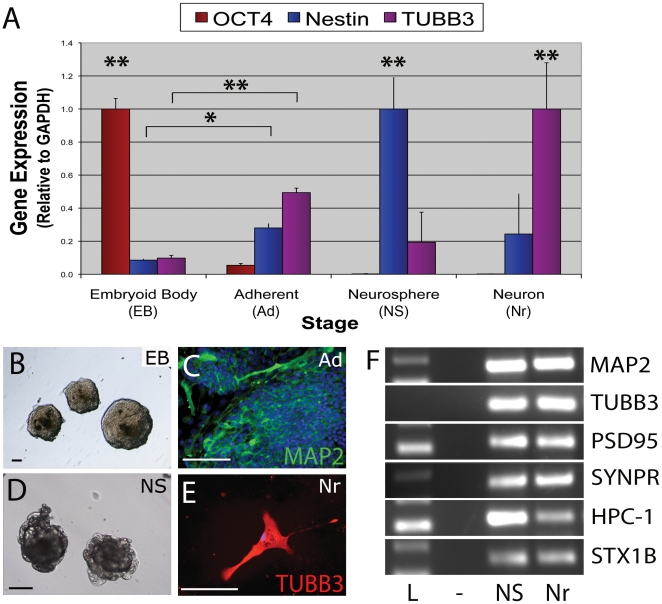
Induced differentiation generates incompetent neural cells from immunodissection-generated cESC lines. Canine ESCs without neural predisposition (OVC.ID lines) produce expected expression profiles during RA-induced differentiation (A–D), but exhibit improper neuronal phenotypes (E) even while expressing mature neuronal genes (F). (A) Upon neural induction, pluripotency gene expression (OCT4) is down-regulated and proper temporal upregulation of early (Nestin) and late (TUBB3) neural genes occurs throughout progressive stages of differentiation. (B) LIF removal allows embryoid bodies (EBs) to form from OVC.ID colonies. (C, D) Treatment with RA and adherence to Fn/ITS substrates induces a high proportion of MAP2+ neural cells, leading to neurosphere (NS) formation. (E) Culture in NT-3/NGF/BDNF-supplemented media allows cells to grow out from NSs. These neural-like cells are sparse, and absent of neuronal processes or interactions, suggesting expression profiles are inadequate to characterize putative progenitor populations. DAPI was used as nuclear counterstain (blue; C, E). Scale bar  =  100 µm. *, p<0.05; **, p<0.01. Real-time RT-PCR data was normalized to highest expression level per gene and calibrated with respect to GAPDH. (E) Lanes: L, molecular weight ladder; -, negative control; NS, neurosphere; Nr, putative neuron. Abbreviations: ESC, embryonic stem cell; Fn, fibronectin; ITS, insulin/transferrin/selenium; RA, retinoic acid.

We subsequently used cESC lines derived by immundissection (OVC.ID) that do not exhibit default differentiation into neural lineages [24] to evaluate the effectiveness of producing competent neural precursor cells through factor-induced differentiation. Using the five-stage RA-based differentiation protocol that was developed before the advent of the neural default pathway [33], [34], we tested the stepwise expression of neural genes and proteins using Real-time qRT-PCR and immunocytochemistry. OCT4 was highly expressed in OVC.ID colonies; however, OCT4 was down-regulated between 5 d embryoid body (EB) and adherent-EB (Fn/ITS plated EB) by 18-fold (p<0.01), and abrogated upon further differentiation (Fig. 2A). Concurrently, stage-specific regulation of neuronal genes mimicked neuronal development with immature (Nestin) and more mature (TUBB3) neural cell types showing upregulation and peak at appropriate time points for neurosphere (NS) and putative neuron (Nr) stages. Differentiating cells also exhibit a high expression of MAP2 and TUBB3 protein from the adherent-EB stage onward (Fig. 2B–E). Mature neuronal cytoskeletal and synaptic genes were similarly expressed in neurospheres and putative neurons, indicating differentiated cells are mature neuronal derivatives (Fig. 2F).

In spite of this, the putative neurons produced by a RA-based induction protocol did not exhibit appropriate processes, cell-cell connections, or dense networks (Fig. 2E). In fact, the putative neurons could not be produced in sufficient numbers to properly perform co-culture and electrophysiology assays.

### Primary astrocytes, neurons, and cESC-derived neurons from explant-generated cESC lines

**Figure 3:**
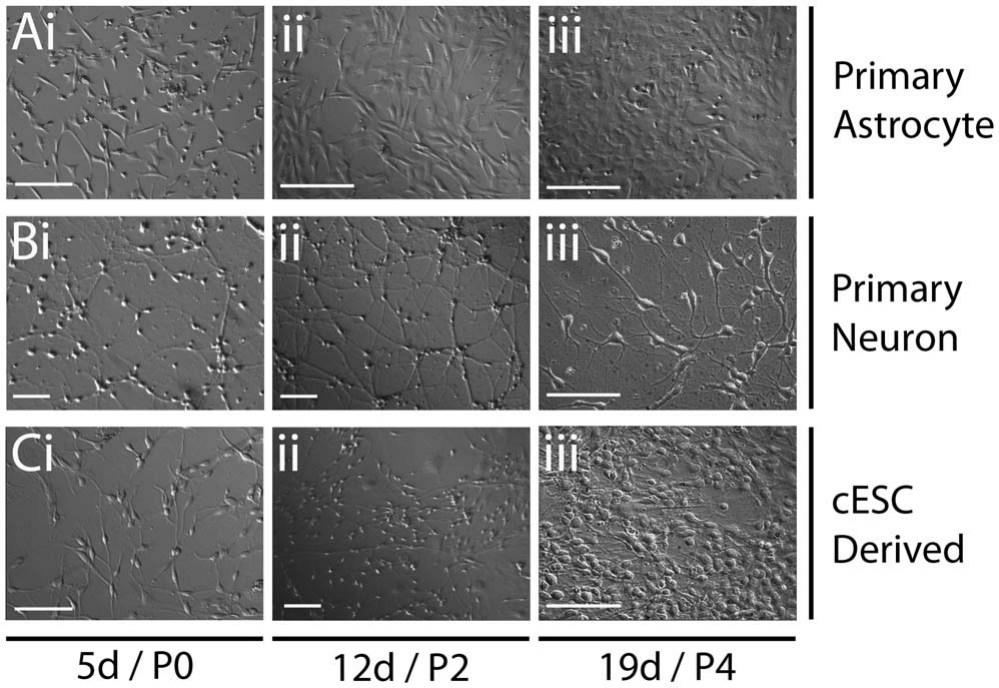
Morphology of primary astrocyte, neuron, and cESC-derived cultures. Development of primary and cESC cultures shows progressive differentiation through stem cell (i), progenitor (ii), and mature (iii) stages. (A) Primary fetal canine cells maintained in 10% serum (ASM) generate confluent astrocyte-like monolayers. (B) Primary cells maintained in N2B27 media without mitogens generate increasing cell specification and complexity of neuron-like cell networks. (C) Canine ESCs maintained in EGF without LIF/bFGF produces outgrowths of radial cells that develop into specialized neuron-like networks. Scale bar  =  100 µm. Abbreviations: ASM, astrocyte selection media; d, day; P, last day of passage indicated.

Primary cell lines were established to model canine neural development and verify cESC differentiation (OVC.EX lines). Primary cerebral cells were isolated from canine fetal whole cerebral tissue at 6 weeks of gestation. Approximately 5×10^4^ cells/cm^2^ were plated on coverslips coated with poly-D-lysine and laminin in media designed to selectively propagate either astroglia (ASM: astrocyte selection media) or neurons (N2B27 [43]). By day 5, spindle-shaped cells with short processes proliferated in ASM, which continued to select predominantly flat, bipolar cells (Fig. 3Ai, ii). Confluence was reached quickly as cell populations formed contiguous, flat monolayers (Fig. 3Aiii). These astrocytes produced confluent layers up to at least P7 after freeze-thaw (n = 3), allowing easy use for feeder layers in co-culture experiments. Culture of dissociated tissue in N2B27 neuronal selection media produced thin and arborized bipolar cells with apparent somas (Fig. 3Bi). These populations were proliferative, and increased in numbers without becoming flattened or astrocyte-like (Fig. 3Bii). Neurons with evident interactions were seen by day 19, which increased in network size and complexity thereafter (Fig. 3Biii).

To establish viable canine neural stem/progenitor cells (cNSC) from cESCs, we adopted a lineage-selection approach. Noggin and EGF block or circumvent BMP signaling, and efficiently direct towards a neural lineage fate in human and mouse ESCs [31], [32], [44], [45], [46]. Canine ESC lines generated from explant cultures (OVC.EX.4, 5, 7) were directed to neural lineage restriction with exposure to 20 ng/mL EGF or 50 ng/mL Noggin while in monolayer culture. After passaging cESC cultures twice in respective treatments, bipolar putative cNSCs were seen in isolation and in colonies (Fig. 3Ci). Proliferation and cell migration of cNSC populations was evident by passage (P)2 (Fig. 3Cii). Accordingly, expanded populations of highly dense neuronal cells possessed long, arborized neuron-like processes with domed somas indicative of neuron specification (Fig. 3Ciii).

### Canine ESC-derived NSC colonies are EGF and NOGGIN responsive

**Figure 4:**
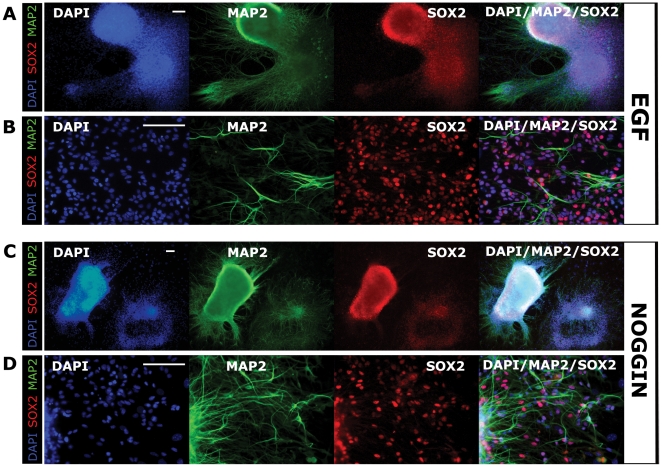
EGF and Noggin are sufficient to generate NSCs from explant-generated cESC lines. Canine neural stem cell (cNSC) colonies are rapidly generated following the addition of EGF (A, B) or Noggin (C, D) and removal of LIF and bFGF. Colonies retain SOX2 expression while exhibiting large outgrowths of differentiating neural cells during lineage specification by EGF (B) or Noggin (D). Cells migrating from colony peripheries obtain MAP2+ neural phenotypes among SOX2+ progenitor populations (B, D). All images were taken at P4. DAPI was used as nuclear counterstain (blue). Scale bar  =  100 µm. Abbreviation: P, passage.

Noggin- and EGF-directed neuralization established similar cNSC colonies from OVC.EX lines by P4, consisting of tightly packed SOX2+ neural stem cells (Fig. 4A, C) with peripheral outgrowths of migrating progenitors (Fig. 4B, D). Upon continued migration from parental colonies, cNSCs revealed increasing neuronal phenotype with multiple MAP2+/TUBB3+ processes and reduced SOX2 expression (Fig. 4B, D; data not shown). Canine NSC colonies were easily maintained upon repeated passaging in monolayers (Fig. 4, 5B). While there were few differences between EGF- and Noggin-treated cNSC lines, EGF groups exhibited less compact colonies with higher outgrowth of compact cells, where Noggin groups exhibited more SOX2+ dense colonies with a higher degree of arborization in migrating progeny (Fig. 4C, D).

### Primary and cNSC lines produce comparable and robust neural derivatives

**Figure 5:**
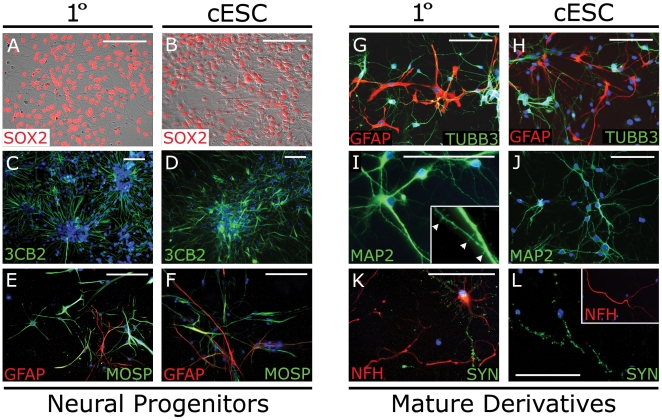
Primary and cNSC lines differentiate into various neural progenitors and mature derivatives. Canine explant-generated ESCs generate neural cells (right columns) equivalent to primary neural progenitors (left columns) when cultured in minimal media supplemented with N2B27, NT-3/BDNF or PDGFαα/T3. (A, B) Bipolar SOX2+ neural progenitor cells quickly form following lineage selection. (C, D) Neural rosettes of 3CB2+ cells form within 1 week. (E, F) Culture in PDGFαα and T3 produce MOSP+/GFAP+ glial progenitors. (G, H) Culture in N2B27 produces mature morphologies of GFAP+ astrocytes and TUBB3+ (Tuj1) neuronal cells. (I, J) Multipolar neurons exhibit extensive branching and MAP2+ dendritic spines (arrowhead, inset). (K, L) Neurons also exhibit mature axons expressing 200 kDa heavy neurofilament (NFH) and punctate synapsin (SYN). Images were taken following culture for 1 week (A–D, G–H) or 3 weeks (E–F, I–L), but all phenotypes were persistent to 6 weeks. DAPI was used as nuclear counterstain (blue, C–L). Scale bar  =  100 µm. Abbreviations: cNSC, cESC-derived canine neural stem cell; MOSP, Myelin/Oligodendrocyte specific protein.

Explant cells from cNSCs (OVC.EX lines) could be maintained in monolayers up to and beyond P6 while retaining SOX2+ expression (Fig. 5A, B) and glia and neuronal potential. To determine differentiation capacity, cNSC lines were taken at P4 and compared to primary fetal cerebral lines grown in equivalent assays. Bipolar progenitor cell aggregates were produced within 1 week that resembled neural rosettes with radial progenitor-like cells [47] that express the radial glia marker 3CB2 (Fig. 5C, D). Radial progenitors persisted only when propagated on MEF feeders in N2B27 media supplemented with bFGF, but could be transiently seen in ASM, or N2B27 without bFGF (data not shown). Primary lines exhibited a more densely packed and elongated rosette structures than cNSC lines. Culturing cNSCs in low-dose PDGFαα/T3 for 3 weeks produced GFAP+ and Myelin/Oligodendrocyte specific protein (MOSP)+ cells consistent with astrocytes and oligodendrocyte precursor cells, respectively (Fig. 5E, F). Culture in N2B27, on the other hand, rapidly produced mixed neural fates, including GFAP+ stellate cells resembling various astrocytes, and neuronal cells with elongated TUBB3+ processes (Fig. 5G, H).

To promote increased maturity of neuronal differentiation, cNSC-derived and primary astrocytes were trypsinized after 3 weeks of initial culture and plated on coated coverslips to confluence. Putative neurons were co-cultured on astrocyte-seeded coverslips and morphologically distinct neuronal-like cell types arose within 3 weeks. Long, thin immature dendritic spines were also seen at this time using MAP2-immunocytochemistry (Fig. 5I, J). Evidence of functional morphological maturation was also seen, as neurons exhibited NFH+ axons that contained synapsin (SYN) positive synaptic vesicles along its length (Fig. 5K, L). Expression of mature axonal and synaptic proteins persisted to 6 weeks, and was present when neurons were grown in astrocyte co-culture with or without addition of NT-3 and BDNF. Canine fetal astrocyte and neuronal cell types could be produced with simple adaptation of mouse- and human-based culture conditions [32], with both primary and cESC-derived lines exhibiting equivalent potency and cell maturity.

### Co-cultured cNSC-derived neurons exhibit mature synaptic physiology

**Figure 6:**
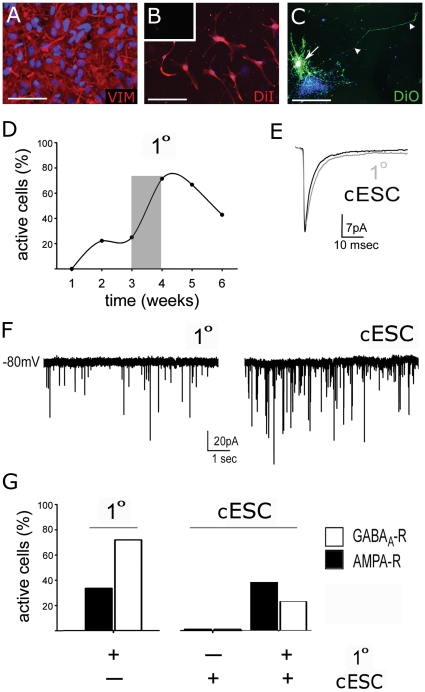
Co-culture on primary astrocytes produces functional cESC-derived neurons. When cultured on confluent layers of astrocytes (A–C), canine stem cell-derived neurons grown with primary fetal neurons received functional inhibitory and/or excitatory synaptic inputs (E–H). (A, B) Confluent layers of canine fetal astrocytes were labeled with DiI membrane dye, which was not visible at 488 nm (inset), and used as substrates for co-culture. (C) Canine ESC-derived cells were labeled with DiO (arrow), which extended along the length of entire cell (arrowhead) within 24 hr, and not to neighbouring cells. (D) Peak number of cells receiving synaptic input was reached at week 4 (characterized in G) with nearly 75% of sampled cells receiving synaptic input. (E) Mean spontaneous excitatory postsynaptic currents in primary and cESC-derived neurons. Basic membrane characteristics and rise-times of synaptic currents were equivalent between cells. (F) Primary and cESC-derived neurons exhibited spontaneous inhibitory synaptic input after one week of co-culture. (G) At 3–4 weeks (shaded in D), primary fetal neurons exhibited predominantly inhibitory GABAergic synaptic activity, while cESC-derived neurons displayed more glutamatergic synaptic activity, almost solely in the presence of primary neurons. DAPI was used as nuclear counterstain (blue, A–C). Scale bar  =  100 µm.

To determine whether cNSC lines generate functional neurons, we used whole cell voltage clamp analysis of spontaneous synaptic events. Canine ESC-derived neurons were cultured with or without primary cerebral neurons on astrocyte feeders for 6 weeks. Feeder layers were confluent, Vimentin+ primary or cESC-derived astrocytes (Fig. 6A). Astrocytes and primary neurons were labeled with Vybrant DiI membrane tag, and cESC-derived neurons were distinguished with Vybrant DiO prior to co-culture (Fig. 6B, C). All cESC-derived and primary neurons displaying synaptic activity exhibited characteristic Na^+^ and K^+^–mediated inward and outward currents when stepped from −100 to 0 mV in voltage-clamp mode (ie. were capable of generating action potentials), while none of the non-active cells displayed these characteristic currents. Inhibitory and excitatory synaptic activity of canine neurons was not evident at one week of co-culture, but increased to ∼75% of recorded neurons by week 4, and declined thereafter (Fig. 6D). Analysis of individual AMPA-R mediated synaptic events in primary neurons alone or cESC-derived neurons co-cultured with primary neurons showed functionally equivalent membrane and synaptic characteristics (Fig. 6E). Spontaneous excitatory and inhibitory currents recorded from primary neurons or cESC-derived neurons co-cultured with primary neurons were similar (Fig. 6F).

Quantitative characterization of synaptic activity at 4 weeks revealed that primary neurons exhibit predominantly inhibitory GABAergic synapses (Fig. 6G). Furthermore, the number of functionally active canine ESC-derived cells was less than observed in primary neurons, and canine ESC-derived neurons exhibited predominantly excitatory glutamatergic synapses. Synaptic activity in cESC-derived neurons was rarely seen in the absence of primary neurons (n = 1/15 individual recordings). These data demonstrate that cells differentiated from cESC-NSCs require a neuron specific supportive niche to fully differentiate into functional neurons and integrate into established neural networks.

## Discussion

Herein, we have presented cESC-derived NSC lines that are capable of differentiating into functional neurons. We have also shown that NSC lines can be derived from cESCs with simple application of EGF or Noggin and maintained at sufficient culture periods for expansion. Using cESC lines that do not exhibit a neural default (OVC.ID), we then showed the RA-based neurosphere differentiation protocol was not able to generate sufficient putative neural cells from OVC.ID lines to test their synaptic physiology, and was greatly inferior to N2B27 differentiation following lineage selection. Definitive proof of this would require RA-based differentiation of OVC.EX lines; however, we have supported this conclusion in a preceding study [24]. Finally, canine NSC and primary fetal neural lines were demonstrated to be effectively equivalent in capacity for deriving cell types throughout each stage of neurodevelopment, from radial progenitor to mature neuron and astrocyte. These results give proof-of-concept that progenitor cell types can be produced in sufficient quantities for use in canine therapeutic cell transplantation for SCI in veterinary medicine.

These results expand upon neural differentiation studies of human and mouse ESC lines and demonstrate that major signaling pathways are indeed strongly conserved in other mammalian species. Early studies of NSCs isolated from the mouse brain as neurospheres used electrophysiology to demonstrate unequivocal neuronal identity and full neural potency [28]. Our study used similar yet updated methods to show unequivocal identity of cNSC-derived cells. Human iPSC/ESCs and murine ESCs are most efficiently driven to neural lineages using Noggin and EGF, respectively [32], [48]. Neural induction can be performed either in classic neurosphere assay or in monolayer culture [27], [31], [33], [49], [50]. Induction using Noggin produced cESC-NSCs and their outgrowths that are quite similar to hESC-NSCs; however, this is not surprising since cESCs share colony morphology and surface antigen characteristics with hESCs (SSEA1−/SSEA3+/SSEA4+). In addition, this is the first demonstration of functional neural cell types derived from canine fetal brains, a potential resource for future studies on canine CNS disease physiology.

The failure of the RA-based protocol on non-neural OVC.ID lines suggests that pluripotent cells—in some if not all species and isolation methods—should not be forced into neural phenotypes with high-dose mitogens to produce cells for transplant. The seemingly appropriate expression profiles produced by RA-based differentiation demonstrate that cellular activity, such as synaptic activity, must be used to verify neural identity. Similar neural phenotype mimicking has been previously shown, where culture in high-dose neural growth factors was able to induce electric waveforms and TUBB3 expression in dermal fibroblasts [51].

Transplanting precursors of myelinating cells is a proven therapeutic strategy to ameliorate damage to the CNS. Robust myelin production with subsequent recovery of sensorimotor function and spinal cord electrophysiology has been demonstrated in numerous mouse, rat and primate models of SCI [1], [39], [40], [41], [52]. Differentiating mature oligodendrocytes could be performed to validate the full gliogenic capacity of cNSC lines; however, cNSC lines were shown to generate mature glia and neurons. The ability of cNSCs to generate synaptically competent neurons was used as a gold standard to test completeness of cNSC potency. The inability of cNSC-derived neurons to produce spontaneous synapses reliably without primary neurons suggests that they require a stronger niche environment to create functional networks. However, successful integration of cNSC-derived neurons in the presence of primary neurons suggests that cNSC lines are capable of integration with host spinal tissue. The propensity of cNSCs to become neurons, astrocytes or oligodendrocytes upon transplantation in the injured cord is more vital, and will require additional study.

Transplanted cells will also require proper labeling to ensure sufficient follow-up. While MR and CT imaging modalities can infer the presence of and response to grafted cells during follow-up [16], [23], [53], self-inactivating lentivectors must be used to label cNSCs with fluorescent proteins prior to cell delivery for confirmation and analysis. Membrane-bound dyes such as DiO/DiI can leach and fade over time, offering another impetus for labeling with reporter proteins.

Lesion volumes resulting from spinal cord injury are incredibly larger in humans than rodents, creating a great barrier to clinical translation. This size disparity is further emphasized in many rodent models of SCI that use thin incisions (hemisection), a problematic tactic often used for studying cell types with lower efficacy [1]. Rodent models are commonly used since the lesions are small and well understood; however, pathophysiology is similarly well known in the dog. Acute SCI due to vehicular trauma and disc herniation is common in the domestic dog, which has resulted in patent knowledge of glial scar formation, histological changes, vascular remodeling, electrophysiology and axonal inhibition [22], [23].

Expert neurosurgical and medical management of SCI is established at several veterinary referral centers. Consequently, clinical trials of therapeutic stem cell delivery following real-world contusion/compression SCI can be conducted that include diagnostic imaging using MR, PET and CT, decompressive surgical intervention, standardized assessment of ambulation, sensation and resolution of neurologic sequelae [54]. This study offers the methodology for scalable generation of cells that could be used in the dog as a phase I/II clinical trial for veterinary medical intervention or prehuman model of cell delivery. These restricted precursors can be cryopreserved and provided as an off-the-shelf product for use in veterinary trauma centers. Furthermore, successful generation of canine (c)iPSCs has been recently reported [55], allowing the potential to create patient-specific neural progenitors for cell delivery models in the dog. These ciPSC lines were shown to express OCT4/ALP and expressed FLK-1, AFP and TUBB3 after rapid differentiation; however, the ciPSC lines still require the proper molecular characterization in concordance with accepted standards within the field [56], [57].

Lineage restriction along the default differentiation pathway efficiently produces neural derivatives from canine ESCs. The NSCs and neurons produced by this method are physiologically active and can be used as a cell source for transplantation in canine SCI. Beyond the inherent value to veterinary medicine, this therapeutic strategy could create valuable clinical extrapolations for human medicine. Medical practice is highly translatable between large animals, as evidenced by the rapid adoption of methylprednisolone for SCI in the dog based on arduous human clinical trials [58]. It follows that safety and efficacy can be clinically determined in large animal patients to inform future clinical trials in human. While this is not a common platform used to infer clinical safety for humans, the costs accrued by Geron Corp (Menlo Park, CA, USA) using traditional preclinical validation suggest that using large animal, non-experimental, clinical platforms may be a much more pragmatic strategy.

## Materials and Methods

### Canine ESC culture

All embryos, fetal tissues and cells were obtained under strict adherence to the guidelines of and approved by the Society for Theriogenology, University of Guelph's Animal Care Committee and Canadian Council on Animal Care. Culture of canine ESCs was performed as previously described [24] from morula and blastocysts stage embryos (Approval No. 07R075). Briefly, cESC lines produced from immunodissection of the ICM (OVC.ID) or explant of whole blastocysts (OVC.EX) were plated on 10^5^ cells/cm^2^ mitotically-arrested murine embryonic fibroblasts (MEF) (ATCC, SCRC-1040) in ESC media that consisted of knock-out (KO)-DMEM, 15% KO serum replacement, 4 ng/mL bFGF (Invitrogen), 10 ng/mL hrLIF (Sigma), 15 µM adenosine, cytidine, guanosine and uridine, 5 µM thymidine, 2 mM L-glutamine, 1× non-essential amino acids, and 0.1 mM beta-mercaptoethanol. Media was replaced by half every 2 days. Canine ESC cultures were passaged every 4–7 days by mechanical dissociation in 30 µg/mL EDTA or 200 U/mL (0.1%) Collagenase IV, and resuspended in a 1∶1 mixture of fresh and conditioned media. For serum-only spontaneous differentiation, cESC colonies from lines OVC.EX were plated onto gelatinized flasks in cESC media, without LIF or bFGF (termed basal media), and with 10% FBS for up to 4 weeks before fixation for ICC staining. Assays were performed on two to three clonal cell lines, immunodissected lines (OVC.ID.20, 21 and 23) for induced RA-based differentiation and explant lines (OVC.EX.4, 5 and 7) for EGF and Noggin lineage selection, neural differentiation, and electrophysiology. No differences were observed between individual OVC.ID lines or OVC.EX lines, and data within groups were pooled. Differences between OVC.ID and OVC.EX lines to differentiation protocols, i.e. inability to patch clamp OVC.ID putative neurons, and inability of OVC.ID cNSC lines to reach P4, resulted in the inability to functionally compare OVC.ID to OVC.ES lines.

### Primary neural cell culture

To obtain fetal brain tissue, two bitches were inseminated and surgical spays were performed at approximately 42 and 43 days of gestation (Approval No. 07R075 under previously stated ethical review boards). Whole uteruses were extracted and placed in PBS on ice for transport. Fetal canine pups were removed by sterile dissection without disturbing the placental ring. Tissues were dissected and snap frozen in liquid nitrogen or placed in dissociation media for cell culture. For primary cell cultures, cerebral tissues were extracted whole using fine forceps with connective tissue (dura and arachnoid maters) and vessels removed. Tissues were digested in dissociation media consisting of 20 U/mL Papain (Sigma) in basal culture media at 37°C for 30–45 min. Following dissociation, cells were plated on 9 cm culture dishes at 4×10^4^ cells/cm^2^ in Neurobasal media supplemented with 1× N2B27 (all from Invitrogen) and 0.5 mM L-glutamine for selection of primary neurons (N2B27), or 10% ESC-qualified FBS, 3 mM L-glutamine for primary astrocyte selection media (ASM).

### Astrocyte-neuron co-culture

Following incubation in ASM for 13 days, astrocytes were trypsinized and plated on 12 mm glass coverslips (Fisher) in 4-well dishes at 4×10^4^ cells/cm^2^ and cultured for 4–6 days to reach confluence. Coverslips were pretreated for adherence with 0.1 mg/mL poly-D-lysine, aspirated and allowed to dry overnight, then coated with 1 ng/mL laminin. To assess neural development in vitro, primary neurons were plated onto astrocyte feeder layers at 5×10^4^ cells/cm^2^ and co-cultured for up to 8 weeks prior to analysis with electrophysiology as described below. Control cultures contained only primary astrocytes or primary neurons. Astrocyte only, neuron only, and astrocyte plus neuron co-cultures were fixed with 2% Paraformaldehyde (PFA) for ICC analysis of protein expression at 1, 3 and 6 weeks.

### Induced differentiation with RA

Using OVC.ID colonies, which do not spontaneously default to the neural lineage, cESCs were directed to differentiate using early neural induction protocols [34]. Culture on non-adherent gelatinized dishes without feeders for 2 days produced embryoid bodies (EB). These EBs were then cultured in basal ESC media with 4 ng/mL bFGF, 10% FBS, and 5 µM Retinoic Acid (Sigma) in non-adherent bacterial culture dishes for 8 days. Following a subsequent 6 day culture in basal media with 1% ITS and 50 µg/mL fibronectin (StemCell Technologies), neurospheres (NS) were observed. To increase NS attachment, 10% FCS was added on the first day only. For differentiation of terminal neurons (Nr), neurospheres were plated at 5×10^5^ cells/cm^2^ on poly-L-ornithine and fibronectin-coated coverslips, and cultured for 14 days in ES Basal Cult with 1× N2-A, 0.2 mM ascorbic acid (StemCell Technologies), with supplementation of 10 ng/mL hrBDNF, 15 ng/mL hrNT-3, and 3 ng/mL hrGDNF supplements (R&D) for the first 4 days. Cells were isolated for quantitative RT-PCR or fixed for staining at stages EB, adherent EB, NS and Nr.

### Neural selection with EGF or Noggin

To determine neural selection potential of cESCs, explant lines (OVC.EX.4, 5 and 7) were grown on MEF feeders in 35 mm or 4-well dishes in basal ESC media with bFGF, without LIF, for 14 days. Canine ESC cultures were then maintained in the presence of 20 ng/mL FGF2 with either 20 ng/mL epidermal growth factor (EGF) or 50 ng/mL Noggin (R&D) [88, 123] for 6 days. Whole dishes were passaged using collagenase up to passage (P)4 and fixed for immunostaining at P1, P2 and P4. To isolate and establish putative canine neural stem cell (NSC) and radial progenitor cell (RPC) in monolayers, Noggin-restricted colonies were isolated and passaged on MEF seeded 4-well dishes in N2B27 media with 20 ng/mL FGF2. In each group, media was replaced every two to three days.

### Directed differentiation of canine NSCs

Canine ESC-derived NSC/RPC cell lines from EGF and Noggin treated groups were taken at P4 and cultured as described above in N2B27 or ASM for differentiation of neurons and astrocytes, respectively. Cells were then fixed and stained at 1, 3 and 6 weeks as described for primary cell lines. Further supplementation of N2B27 media with 30 ng/mL NT-3 and 100 ng/mL BDNF was performed for three groups, however no differences were seen in morphology, protein expression or electrophysiological properties, and data were pooled. For differentiation of oligodendrocyte precursor cells (OPCs), primary fetal neural stem cells and Noggin-restricted cESC-derived NSCs at P4 were cultured in N2B27 media supplemented with 20 ng/mL bFGF and 10 ng/mL PDGFαα (Peprotech) for 8 days, with 30 ng/mL T3 (Sigma) [59] for an additional 6 days. Cells were fed and fixed as above.

### Immunocytochemistry

Cells and neural derivatives were fixed in 4% or 2% paraformaldehyde, respectively, for 15 min at room temperature (RT) and washed 3 times in PBS. Embryoid bodies were fixed, washed, maintained in 30% sucrose, snap frozen in OCT and sectioned on a cryostat at 12 um thickness. Blocking and permeabilization was performed with 0.1% Triton-X-100 and 10% normal donkey serum (NDS) or 10% normal goat serum (NGS) for 45 min at RT. Cultures were incubated at 4°C overnight with primary antibodies (Table S1) diluted in 10% NDS or 10% NGS, 0.01% PBST (Tween-20 in phosphate-buffered saline), then washed three times for 15 min with 0.1% PBST. Appropriate secondary antibodies (1∶500, all AlexaFluor from Molecular Probes) were then incubated in 10% blocking solution for 1 hr at RT. Nuclei were counterstained with 1 ng/mL DAPI in PBST for 5 min, followed by three washes. Detergents (Triton-X-100, NP40, Tween-20) were not included when staining against cell surface antigens. Primary antibodies were replaced with PBS for negative controls in each round of staining. Imaging was performed on an Olympus X71 epifluorescent microscope using Q Capture software (QImaging). Any and all alterations to global brightness or contrast were first made to secondary-only control files of equivalent imaging parameters to verify their acceptability. Images were compiled and merged using Adobe Photoshop CS3.

### RT-PCR

All RT-PCR was performed as previously described [24]. Briefly, total RNA from whole dishes or floating spheres was isolated with RNeasy Micro kits (QIAGEN) according to manufacturer's directions using on-column DNase I digestion and gDNA Eliminator Spin columns (QIAGEN). cDNA libraries were generated using Superscript II First-strand systems (Invitrogen) in triplicate, including RT omitted controls, and with 1 uL of 100 ng/mL RNase A digestion. RNA/cDNA quality and quantity was determined on a ND-1000 (NanoDrop Technologies). Intron-spanning or flanking primer sets were designed using Primer3 online software (http://frodo.wi.mit.edu/) and Perl Primer (http://perlprimer.sourceforge.net). Primers were used only if a single amplicon of expected size were obtained, with sequencing used to verify gene-specific identity (Table S2). Expression profiles were examined at multiple stages including: 5 day EBs (EB), adherent EBs at start of ITS/Fn treatment (Ad), neurospheres (NS), and putative neurons (Nr). Amplification consisted of 27 or 30 cycles, found to be within linear range for all data, and negative controls remained signal null until cycle 40.

Quantitative gene expression was determined with Real-time qRT-PCR on a LightCycler 1.5, analyzed with 4.0 Exor software (Roche). Gene-specific primer design and cDNA library creation were performed as above. Reactions were performed with SYBR green I DNA master mix (Roche) in 10 µL volumes. All Real-time data was collated and corrected according to the Pfaffl-based ΔΔCp method [60], [61], [62] with relative expression  =  E_(t)_∧[Cp_(t)_(target-calibrator)]/E_(r)_∧[Cp_(r)_(reference-calibrator)]. Three samples of each stage were analyzed in triplicate. Samples were normalized to and validated with GAPDH, RPS18, and RNA Pol II as housekeepers, with final data presented relative to GAPDH [13].

### Electrophysiology

For electrophysiological analysis of synaptic activity, five groups of cells were co-cultured on primary astrocyte feeder layers: primary neurons; Noggin-treated cES cells with and without primary neurons; EGF-treated cES cells with and without primary neurons. Co-culture was performed on confluent astrocyte feeder layers as described above. Primary cells and astrocyte feeders were labeled with DiI (red; V-22885, Molecular probes), and cESC-derived neurons were labeled with DiO (green; V-22886; Molecular probes) to allow impartial distinction of cESC-derived and primary cells. Whole cell patch-clamp recordings of primary neurons or cES cells were performed as previously described [63]. Coverslips were transferred to recording chambers where they were continuously perfused with aCSF and visualized using infrared DIC microscopy combined with epi-fluorescent. Tetrodotoxin (TTX) was added to perfusion media while the cells were held at −80 mV in voltage-clamp mode. To measure GABA_A_-R mediated events, cells were gradually stepped to a holding potential of 0 mV and spontaneous synaptic activity was recorded for 5 min. Following a 2 min wash-in period GABA_A_-R mediated synaptic events were completely blocked by perfusion of 10 uM bicuculline (Bic) and 50 uM picrotoxin (Pic). Cells were then stepped to −80 mV, allowed to recover for several minutes, and spontaneous AMPA-R mediated synaptic event were recorded for 5 minutes. All excitatory synaptic activity recorded in BicPic was inhibited using the AMPA/kainite receptor antagonist NBQX (20 um). Network burst activity of spontaneous polysynaptic AMPA-R mediated events was recorded at −80 mV in BicPic without TTX in separate slices. Each recording was analysed for the frequency, inter-event interval and amplitude of AMPA- and GABA_A_-mediated synaptic currents. A single author (JKYL) blinded to the measured cell types confirmed currents and tracings. Measurements that had capacitive transient less than 1000 pA or access resistance that varied by 20% as measured during a 10 mV voltage-step uncompensated recording were excluded. Due to the conditions of co-culture and presence of multiple cell types, unbiased techniques of cell selection for electrophysiology could not be used. Consequently, we report the activity of cells as a percentage of all cells recorded from, as described. No difference was seen between Noggin- and EGF-treated groups, and data were pooled.

### Statistical analyses

Analyses were performed using Microsoft Excel software (Microsoft, Seattle WA) and statistically analyzed using SAS software (SAS Institute Inc, Cary CA). Quantitative Real-time RT-PCR was analysed for significance with normalized, calibrated expression ratios determined by one-way ANOVA statistical test with Tukey and Tukey-Kramer post hoc multiple comparison algorithms. Heterogeneous data sets (typically with values <0.01) were transformed prior to analysis. Electrophysiological results were analysed with non-parametric Kruskal-Wallis one-way ANOVA by ranks (p<0.05), followed by Kruskal-Wallis or Chi-square multiple comparisons (p<0.05) and/or the Kolmogorov-Smirnov test for differences between distributions (p<0.05). Results are stated in mean +/− SEM: *, p<0.05; **, p<0.01.

## Supporting Information

Table S1 **Antibody detail and canine-specific immunoreactivity.**
(PDF)

Table S2 **Primer sequences and details for canine-specific RT-PCR.**
(PDF)
