# Supplementary material for: Synaptically-Competent Neurons Derived from Canine Embryonic Stem Cells by Lineage Selection with EGF and Noggin
Source: PLoS One. 2011 May 17;6(5):e19768. doi: 10.1371/journal.pone.0019768 (PMC3096636; doi:10.1371/journal.pone.0019768)
Supplement: Table S1 — Antibody detail and canine-specific immunoreactivity. (PDF) [file pone.0019768.s001.pdf]

Table S1: Antibody detail and canine-specific immunoreactivity

Antibody	***Company***	***Catalogue***	***Host***	***Dilution***	***Localization***
OCT4	Santa Cruz	SC9081	Rb	1:500	Nucleus
OCT4	Santa Cruz	SC8628	Gt	1:400	Nucleus
SOX2	Stem Cell Tech	1438	Rb	1:400	Nucleus
SSEA-3	Chemicon	MAB4303	Mo	1:250	Surface
SSEA-4	Chemicon	MAB4304	Mo	1:250	Surface
TRA-1-81	Chemicon	MAB4381	Mo	1:250	Surface
FGFR1	Chemicon	MAB125	Mo	1:250	Surface
Vimentin	Chemicon	AB5733	Chk	1:2000	Cytoplasmic
Vimentin	Chemicon	MAB3400	Mo	1:500	Cytoplasmic
3CB2	DSHB	3CB2	Mo	1:500	Cytoplasmic
MOSP	Chemicon	MAB328	Mo	1:500	Cytoplasmic
GFAP	Chemicon	AB5804	Rb	1:2000	Cytoplasmic
GFAP	Chemicon	MAB360	Mo	1:2000	Cytoplasmic
TUBB3 (Tuj1)	Sigma	T8660	Mo	1:2000	Cytoplasmic
MAP2ab	Chemicon	MAB378	Mo	1:5000	Dendrite
MAP2	Chemicon	AB5622	Rb	1:2000	Dendrite
NF-H	Chemicon	AB5539	Chk	1:500	Axon
Synapsin	Synaptic Sys	106-001	Mo	1:100	Synaptic
DiO (Vybrant)	Molecular probes	V-22886	n/a	1:200	Membrane
DiI (Vybrant)	Molecular probes	V-22885	n/a	1:200	Membrane
Not Reactive to Canine Cells					
Nestin	BD Pharmingen	611658			
Nestin	DSHB	RAT-401			
Radial Glia	DSHB	RC2			
Olig	DSHB	Olig			
RIP	DSHB	RIP			
O4	Neuromics	MO15002			
VGlut2	Chemicon	AB5907			
Synaptotagmin	DSHB	Synaptotagmin			
PSD95	NeuroMab	K28/43			

Abbreviations: Chk, chicken; Gt, goat; Mo, mouse; Rb, rabbit.
