# Supplementary material for: Synaptically-Competent Neurons Derived from Canine Embryonic Stem Cells by Lineage Selection with EGF and Noggin
Source: PLoS One. 2011 May 17;6(5):e19768. doi: 10.1371/journal.pone.0019768 (PMC3096636; doi:10.1371/journal.pone.0019768)
Supplement: Table S2 — Primer sequences and details for canine-specific RT-PCR. (PDF) [file pone.0019768.s002.pdf]

Table S2: Primer sequences and details for canine-specific RT-PCR

Gene	*Sequence**	*Size*	*E*
RNA Pol II	GACGAGAAAGGCTTGGTCAG<br>ATGGGTAGGCTTGGAGAGGT	192	1.95
GAPDH	GCCCTCAATGACCACTTTGT<br>TCCTTGGAGGCCATGTAGAC	101	1.92
RPS18	ACTGAGGATGAGGTGGAACG<br>ACACGAAGTCCCCAAAAGTG	215	1.92
OCT4	AGTGAGAGGCAACCTGGAGA<br>TCAGGGAGAGGGACTGAGGA	303[a]	1.89
Nestin	CAGCAGCTAGCACACCTCAA<br>GCAAGGAGAGGGAAGTAGGG	220[a]	1.77
TUBB3	AGCCAAGTTCTGGGAAGTCA<br>CCCACTCTGACCAAAGATGAA	238	1.93
GFAP	AGATCCACGATGAGGAGGTG<br>TCTTAGGGCTGCTGTGAGGT	104	
MAP2	AGAGGAGGTGTCTGCAAGGA<br>GTGATGGAGGTGGAGAAGGA	161	
PSD95	GACGGGAGTGGTCAAGGTTA<br>GGCGAGCATAGTGAACTTCC	120	
HPC-1 (STX1A)	AGTACAACGCCACACAGTCG<br>GTTCCCACTCTCCAGCATGT	122	
STX1B	CAACAAGGTTCGGTCCAAGT<br>ACTGGGTCGCGTTATATTCG	158	
Synaptoporin	GTTGGTGGGTTCATCAGCTT<br>CCAAAGACCACGGAAGTGTT	165	
MBP	AGAAGAGCAACAAGGCTGGA<br>TTGTTCTGCTCCACATCTGC	124	
NEFH	CTCAAAGGCACCAAGGACTC<br>CAAAGCCAATCCGACATTCT	244	
Synaptophysin	GCCACTGACCCAGAGAACAT<br>TCCTTGAACACGAACCACAG	167	

** Primer sets are written 5' to 3'; forward, top; reverse, bottom.*
[a] Elongation time, 20 sec.
Abbreviaitons: E, efficiency of primer set.
